# Education and training of surgical residents in upper gastrointestinal surgery: a European survey

**DOI:** 10.1007/s13304-025-02362-3

**Published:** 2025-08-16

**Authors:** Călin Popa, Diana Schlanger, Alberto Aiolfi, Moustafa Elshafei, Dimitrios Theodorou, Ognjan Skrobic, Aleksandar Simic, Suzanne Gisberz, Davide Bona, Luigi Bonavina, Education Training in Foregut Surgery (ETFS) European Collaborative Group, Emanuele Abate, Ahmed Abdelsamad, Florin Achim, Rita Alfieri, Andrea Balla, Yves Borbely, Nicholas Boyle, Giuseppe Brisinda, Piero Giovanni Bruni, Waqas T. Butt, Matteo Calì, Giacomo Calini, Valentin Calu, Francesco Cammarata, Giampiero Campanelli, Carlo Castoro, Marta Cavalli, Alexandros Chamzin, Mircea Chirica, Serge Chooklin, Nicola Cillara, Michael de Cillia, Sandra Cristina, Fabrizio D’Acapito, Elke Van Daele, Giorgio Dalmonte, Verdi Daunia, Sara De Bernardi, Viorel Dejeu, Lieven Depypere, Octavian Enciu, Félix Ferraz, Gilberto Martinho Santos Figueiredo, Edoardo Forcignanó, José Freire, Uberto Fumagalli, Francesca Gatti, Mircea Gheorghe, Andrei Cristian Ghioldiş, Mario Giuffrida, Ines Gockel, Ewen A. Griffiths, Peter Grimminger, Caroline Gronnier, Christian Gutschow, Anis Hasnaoui, Till Heidsiek, Mark Ivo van Berge Henegouwen, Sandra Hilário, Petre Hoara, Argyrios Ioannidis, Orestis Ioannidis, Stylianos Kapiris, Clive Kelty, Dagmar Kollmann, Dimitris Korkolis, Ivan Kristo, Stylianos Kykalos, André Lázaro, Thorsten Lehmann, Jonathan Levy, John Lipham, Francesca Lombardo, Michele Manara, Luigi Marano, Sheraz Markar, Manuel Mega, Stefan Monig, Emanuele Morandi, Francesk Mulita, Beat Peter Muller, Martina Novia, Fabio Massimo Oddi, Matthias Paireder, Roberto Peltrini, Enrico Pinotti, Mauro Podda, Razvan Popescu, Dragos Predescu, Elisa Reitano, Martin Riegler, Riccardo Rosati, Dimitrios Schizas, Francisco Schlottmann, Sebastian Schoppmann, Galyna Shabat, Natalia Sipitco, Maria Sotiropoulou, Giovanni Domenico Tebala, Flavio Tirelli, Elena Adelina Toma, Tania Triantafyllou, Yener Uzunoglu, Hanne Vanommeslaeghe, Milan Veselinović, Wah Yang, Jörg Zehetner

**Affiliations:** 1 University of Medicine and Pharmacy Iuliu Hatieganu Cluj-Napoca, Regional Institute of Gastroenterology and Hepatology O. Fodor Cluj-Napoca, Croitorilor 19-21, 400162 Cluj-Napoca-Napoca, Romania; 2 General Surgery, IRCCS Ospedale Galeazzi-Sant’Ambrogio, Milan, Italy; 3 Krakenhaus Nordwest, Allgemein-Viszeral- Und Minimal Invasive Chirurgie, Frankfurt, Germany; 4 Hippokration General Hospital University of Athens, Athens, Greece; 5 Department of Esophageal and Gastric Surgery, University Clinical Centre of Serbia, University of Belgrade, Belgrade, Serbia; 6 Department of Surgery, Amsterdam UMC, Amsterdam, The Netherlands; 7 Cancer Center Amsterdam, Cancer Treatment and Quality of Life, Amsterdam, The Netherlands; 8 Division of General and Foregut Surgery, University of Milan, IRCCS Policlinico San Donato, San Donato Milanese (Milano), Milan, Italy

**Keywords:** Surgical training, Medical education, Upper gastrointestinal surgery, Fundoplication, Gastrectomy, Esophagectomy

## Abstract

Educating and training young surgeons is a complex challenge that requires a structured pathway. Upper gastrointestinal (UGI) surgery is becoming a highly attractive subspecialty, yet it is not universally recognized. Currently, there are no standardized guidelines or curricula for UGI surgical training across Europe and a wide disparity among countries remains. This study aims to focus on the current European landscape from the perspective of both trainees and trainers, and to provide a foundation for developing effective guidelines for education and training of surgical residents. We conducted a cross-sectional survey study with targeted questions about various aspects of managing patients with UGI diseases, including preoperative investigations, surgical interventions, interventional endoscopy, and postoperative follow-up. We also assessed the availability of training resources and extracurricular activities. Distinct questionnaires were distributed independently to trainees and trainers. The overall response rate was 35.6%. Most trainees and trainers recognized the need for dedicated courses in UGI surgery and emphasized the importance of learning through video materials. The survey revealed that surgeons are often unfamiliar with UGI endoscopy and functional esophageal function studies. We analyzed residents’ direct involvement in three surgical procedures: anti-reflux surgery, gastrectomy, and esophagectomy. Residents expressed a lack of confidence in executing certain surgical steps. Both trainers and residents expressed preference for extracurricular, hands-on courses focused on UGI surgery but reported insufficient opportunities for such training. This survey highlights significant gaps and limitations of UGI training for surgical residents in Europe. Efforts toward standardization and development of specific guidelines and recommendations are required to enhance education in UGI surgery.

**Supplementary Information:**

The online version contains supplementary material available at 10.1007/s13304-025-02362-3.

## Introduction

The education and training of general surgery residents is complex and challenging due to the rise of subspecialties and the ongoing advancements in technologies, techniques, and surgical innovations [1]. A standardized approach to education and training could enhance the quality of surgical care across Europe and facilitate more effective exchange programs among countries. Traditionally, a strong mentor–trainee relationship has been the primary model for training new surgeons. However, as surgical subspecialties have developed and new diagnostic and therapeutic technologies have emerged, a more intricate infrastructure involving various medical specialists is necessary to deliver effective and targeted surgical treatment. Currently, there are numerous educational tools available for surgical training, including simulation training, hands-on courses, residential programs, webinars, and conferences [2–4]. It is essential that these resources are used effectively to maximize the success of educational programs. Surgical education should encompass the entire clinical process including preoperative diagnostic assessment, selection of the most appropriate surgical procedure, management of complications, and postoperative follow-up. Unfortunately, training often emphasizes nuances of the surgical procedure itself while neglecting other critical aspects of management.

Upper gastrointestinal (UGI) surgery is becoming a significant subspecialty due to the increasing number of patients with both benign and malignant conditions requiring complex interventions. At present, there are no European guidelines specifically addressing the education and training of residents in UGI surgery. Before developing these guidelines, it is crucial to assess the current landscape to understand the perspectives of both trainees and trainers. Our goal was to gather this information through a survey study to identify and address key issues that may impact the education and training process in UGI surgery.

## Methods

This is a cross-sectional survey study addressing the topic of education and training of surgical residents in UGI surgery.

### Planning phase

The timeline of this study, the distribution of the survey, as well as the topics to be covered were planned in June 2023 by a panel of discussants (LB, DT, AS, OS, ME, CP). Several revisions of the survey were afterwards discussed online until a consensus was reached in July 2023.

### Drafting the survey

Two independent surveys were designed to assess the opinions of both the trainers and the trainees [Supplementary Files 1–2]. The surveys were constructed in a Google format for easy distribution and data gathering. A scale from one (poor) to five (excellent) was used for grading the responses.

The main topics of investigation and discussion were the following:

1. Preoperative patient’s assessment
2. Surgical procedures with focus on anti-reflux surgery, gastrectomy, and esophagectomy
3. Interventional endoscopy
4. Postoperative follow-up
5. Available resources for complementary training and extra-curricular activities

### Survey distribution

Participants were recruited through social media platforms (LinkedIn and Twitter), email, and personal invitations. The electronic questionnaire was tested for functionality and was made available online using Google Forms (Google LLC, Mountain View, California, US). The European Foregut Society (EFS) endorsed the project and assisted in disseminating the survey. The survey was further promoted and discussed during the EFS Congress held Milan, Italy, November 2023.

### Data collection, data analysis, and reporting

The data collection took place between July 2023 and December 2023. The responses to the survey were gathered in a Microsoft Excel document, and descriptive statistics were performed. Data were analyzed by the working group (LB, SG, DT, AS, OS, ME, CP, DS) and reported based on the CROSS checklist [5]. No Institutional Review Board (IRB) approval or written consent was necessary for this study.

## Results

### The trainee’s point of view

#### General information

Overall, 117 residents from 14 European countries responded to the survey (Table 1). Median age was 30 years, and 59.8% were males. Most participants (67.5%) received their training in academic hospitals, and about two-thirds (65.8%) were in their last 3 years of residency. The majority of respondents (58.1%) had the opportunity to complete their training in a dedicated UGI center. Overall, 92.1% of participants rated strongly the need for structured courses dedicated to UGI surgical unit. Further, questions were asked regarding the availability of materials and opportunities complementary training in UGI surgery (Table 2**)**.

**Table 1:** Country of origin of trainees who participated in the survey. Data are presented as percentages

Country	%
Italy	34.2
Romania	24.8
Greece	12
France	6
Moldova	5.9
Germany	4.3
Portugal	3.4
UK	1.9
Switzerland	1.7
Serbia	1.7
Netherlands	1.7
Austria	0.8
Hungary	0.8
Spain	0.8

**Table 2:** The availability of resources in the trainee’s opinion. A scale from 1 to 5 was used for grading the responses. Poor: 1–2; moderate 3; good/excellent 4–5. Data are presented as percentages

	Poor	Moderate	Good/excellent
Rate the quality of the available study materials	30.7	36.7	32.6
Do you have available video materials?	41.9	27.3	30.8
Do you have available structured theoretical courses?	47.8	30.8	21.4
Overall, do you feel like there are enough materials available?	28.1	33.3	38.6

#### Preoperative diagnostic investigations

Overall, 53% of participants indicated that they are regularly engaged in preoperative investigations (Table 3). Among the surveyed residents, 73.5% reported that they have never independently performed UGI endoscopy, while 86% have never performed esophageal manometry or pH-impedance studies.

**Table 3:** The resident’s practice in endoscopy, manometry, and pH metry. Data are indicated as percentages

	No	Sometimes	Yes
Have you performed pH/impedance testing?	86.3	4.3	9.4
Have you performed esophageal manometry?	84.6	5.9	9.4
Have you performed upper endoscopy?	73.5	10.2	15.4
Do you have any possibility to get dedicated training in pH/impedance testing?	77.8	14.5	7.7
Do you have any possibility to get dedicated training in esophageal manometry?	76.1	16.2	7.7
Do you have any possibility to get dedicated training in upper endoscopy?	47.8	28.2	23.9

#### Interventions/postoperative follow-up

Overall, 67.5% of residents had never performed an UGI intervention independently, and 32.5% of participants performed at least one procedure. The median number of interventions per resident was 5. Notably, 76.3% of the trainees performed these operations during the last 3 years of residency. The survey also examined residents’ perceptions of the surgical steps involved in three specific procedures: anti-reflux surgery, gastrectomy, and esophagectomy.

Only a small percentage of responders reported having independently performed one or more surgical steps in anti-reflux surgery. Specifically, 24.8% performed crural dissection, 25.6% gastric fundus mobilization, and 20.5% partial fundoplication. Crural dissection and total fundoplication were identified as the most challenging steps by 25.6% and 23.1% of respondents, respectively. In the case of gastrectomy, lesser curvature dissection, greater curvature dissection, and digestive tract reconstruction after subtotal gastrectomy were performed autonomously by 32.4%, 42.7%, and 30% of participants, respectively. Lymphadenectomy and esophagojejunal anastomosis were deemed the most challenging surgical steps by 64.1% and 32.5% of participants, respectively. Regarding esophagectomy, it was reported that gastric mobilization and gastric conduit creation were carried out independently by 17.1% and 7.7% of responders, respectively. Thoracic and cervical esophageal dissection were recognized as the most challenging surgical steps by 35.1% and 28.2% participants, respectively. Data reporting the involvement of residents in postoperative course, management of complications, and follow-up after hospital discharge are depicted in Table 4.

**Table 4:** The involvement of trainees in the follow-up period of patients. Data are indicated as percentages

	No	Sometimes	Yes
Are you involved in the early postoperative follow-up?	5.1	5.2	89.7
Are you involved in the early (ICU) postoperative follow-up?	26.5	13.7	59.8
Are you involved in the late postoperative follow-up?	13.7	27.3	58.9
Are you involved in the treatment of postoperative complications (endoscopic)?	49.5	23.9	26.5
Are you involved in the treatment of postoperative complications (surgical)?	5.4	9.2	85.4

#### Extracurricular activities

Most residents (61.5%) participated in extracurricular activities. Overall, 51.3% of participants stated to be directly involved in research projects including literature review and writing manuscripts for publication. When asked about their preferred types of extracurricular or scientific activities, hands-on training was mostly favored (92.3%), followed by meetings (45.3%), webinars (43.6%), live surgery (67.5%), and residential fellowships (61.5%). Notably, 70.9% of participants preferred international over national events. Regarding hands-on workshops, 76.1% expressed preference for dedicated stand-alone events rather than those associated with meetings. Further, 80.3% of participants preferred live animal surgery.

### The trainer’s point of view

#### General information

**Fig. 1:**
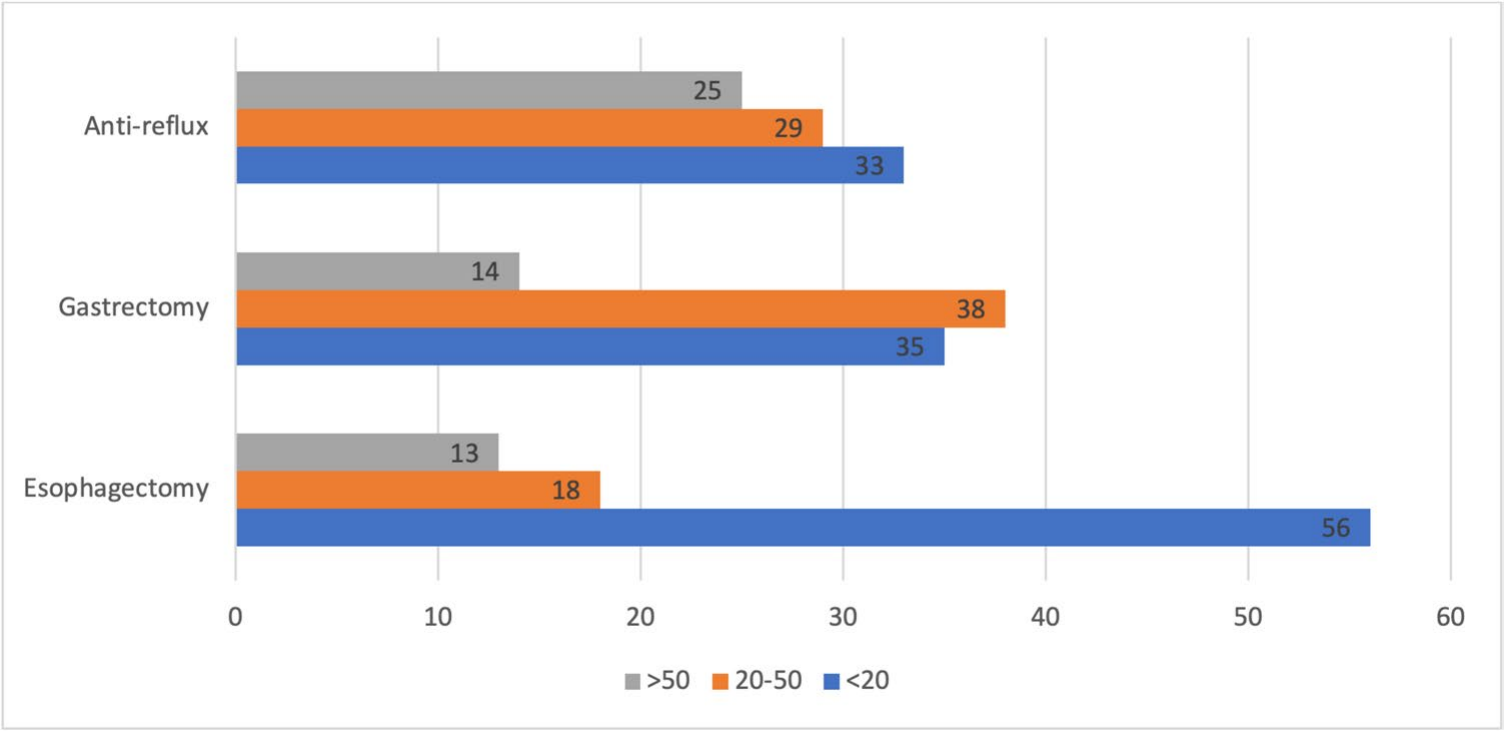
The number of upper GI surgical interventions performed in the trainer’s center per year

Overall, 90 responders from 18 European countries were included (Table 5). The median age was 59.3, and 82.2% were males. Most responders (77.8%) work in an academic hospital. Forty-four (48.9%) of responders work in a dedicated UGI surgical unit. The number of UGI interventions performed per year and the availability of resources are depicted in Fig. 1.

**Table 5:** Country of origin of trainers who participated in the survey. Data are presented as percentages

Country	%
Italy	24.4
Romania	14.5
Greece	16.6
Portugal	7.8
Germany	6.7
France	4.5
Serbia	4.4
Turkey	3.3
Belgium	3.3
Austria	2.3
Netherlands	2.2
Switzerland	2.2
UK	2.2
Ireland	1.2
Moldova	1.1
Poland	1.1
Ukraine	1.1
Spain	1.1

#### Preoperative diagnostic investigations

When asked whether their residents have the opportunity to assist in the preoperative management of the patient, 73.3% responded yes, 17.8% responded sometimes, while 8.9% responded no. The majority (> 80%) of trainers stated that their residents do not get any involvement or dedicated training in esophageal manometry and pH-impedance studies. However, the collaboration with gastroenterologists in multidisciplinary board discussions remains efficient. Overall, 77.7% of trainers have dedicated endoscopic programs in their facilities. The trainer’s attitude on the involvement of residents in operative endoscopic procedures is depicted in Table 6.

**Table 6:** Extracurricular activities: the opinion and involvement of the trainee. Data are presented as percentages

	No	Sometimes	Yes
Do your residents have the possibility to train/practice on rigid endoscopy for the treatment of Zenker’s diverticulum?	85.6	12.2	2.2
Do your residents have the possibility to train/practice on flexible endoscopy for the treatment of Zenker’s diverticulum?	83.3	12.2	4.5
Do your residents have the possibility to train/practice on POEM?	83.3	11.1	5.6
Do your residents have the possibility to train/practice on stricture dilation?	55.5	20	24.5
Do your residents have the possibility to train/practice on stent placement?	60	17.8	22.2
Do your residents have the possibility to train/practice on endoscopic vacuum therapy?	67.8	14.4	17.8
Do you use intraoperative endoscopy for anastomosis verification?	61.1	23.3	15.6
Do you use intraoperative endoscopy for myotomy verification during achalasia surgery?	41.1	22.2	36.7
Do you use intraoperative endoscopy for anti-reflux valve flap verification during hiatal hernia surgery?	51.1	28.9	20

#### Surgical interventions/postoperative follow-up

When looking at anti-reflux surgery, respondents indicated that the hardest surgical step to teach was crural dissection (42.3%), followed by gastric fundus mobilization (36.6%). For gastrectomy, most participants found lymph node dissection (71.1%) and digestive tract reconstruction after total gastrectomy (63.3%) to be the most challenging steps to teach. For esophagectomy, the most difficult steps to teach were thoracic esophagus dissection (61.1%) and cervical esophagus dissection (52.2%), followed by the anastomosis (48.9%). The trainer’s perception on residents’ postoperative and patients’ follow-up involvement indicates a high percentage of residents involved in both early (98.8%) and late (72.2%) postoperative follow-up.

#### Extracurricular activities

More than 80%of trainers reported that their residents have participated in at least one dedicated UGI extracurricular activity in the last year. The types of activities preferred by trainers for their residents were as follows: hands-on training (80%), attending congress/conference (73.3%), fellowships (66.7%), live surgery (50%), and webinars (43.3%).

## Discussion

The education of young surgeons is a critical issue that requires serious attention and support in today’s rapidly evolving field of UGI surgery [6–8]. General surgery residents should gain familiarity with esophageal and gastric surgery and should have the opportunity to pursue this subspecialty during their careers. This necessitates training in preoperative patients’ assessments, therapeutic decision-making, patient selection for surgery, surgical techniques, and postoperative care. To achieve these goals, a structured curriculum is essential. Unfortunately, the diversity in surgical education and training across Europe has not been adequately addressed so far. Our survey aimed to gather insights from both trainees and trainers to provide an overview of current practices and identify potential gaps and solutions. In comparison to previous surveys, this study provides a snapshot of training in UGI surgery throughout Europe, focusing in various aspects of patient management and extracurricular activities [9–11].

We collected 207 responses from 117 trainees and 90 trainers across 18 European countries. Both trainees and trainers shared some concerns about the scarcity of complementary training, suggesting that online and video resources could be particularly beneficial especially in low-volume centers. Neither UGI endoscopy nor esophageal functional studies are integrated into the routine surgical practice of the residents. In contrast, multidisciplinary discussions with gastroenterologists were reported to be generally effective. Regarding interventional endoscopy, most residents lack training opportunities despite being often involved in managing postoperative complications. Only about 20% reported being able to perform interventional endoscopic procedures such as esophageal stent placement, stricture dilation, and EndoVac placement.

Both trainers and trainees were asked about the steps of various surgical procedures, the difficulties encountered, and the most appropriate tasks for independent performance. Interestingly, while 67% of trainers report that residents perform as first-hand surgeons in their practice, only 32.5% of residents agree. Also, crura dissection and gastric fundus mobilization are the steps residents feel confident to perform, and over 50% of trainers are willing to allow residents to take on these tasks. For gastrectomy, both trainers and trainees consider lymphadenectomy as the most challenging step, followed by digestive tract reconstruction after total gastrectomy. The most commonly performed steps by residents are greater and lesser curvature dissection, and reconstruction after subtotal gastrectomy. Similar patterns are observed in esophagectomy, where thoracic and cervical esophagus dissections, as well as the reconstruction phase, are considered difficult by both trainers and trainees. However, only 25% of residents feel confident in performing esophageal dissection, gastric mobilization, and fashioning of the gastric conduit.

Overall, a clear pattern emerged from this survey study. Trainers tend to be more permissive in allowing residents to perform certain steps, while residents lack confidence, possibly due to inadequate experience or lack of support and encouragement from trainers. The absence of a standardized curriculum and progressive training approach may contribute to this ambiguity and insecurity. Given that most surveyed residents are in their final residency years, the lack of confidence in performing basic surgical steps of UGI surgical procedures is concerning and signals the urgent need for a reform in education and training. Trainers should encourage residents’ participation in all aspects and phase of patient’s management at an early stage of the residency program [12]. While most trainers seem to encourage residents to engage in extracurricular activities, only one-third of trainees feel supported in this complementary training also because of the limited offer of dedicated training opportunities. Since hands-on training emerged as a preferred extracurricular activity, European surgical societies and University medical centers should promote and organize more of these events, ensuring equitable access to quality training for residents. The present survey identified the urgent need for a structured curriculum in UGI surgery as an essential complement to existing theoretical resources. Creating a library of instructional videos could help to fill identified gaps and allow trainees to understand complex interventions even in low-volume centers with no specialized UGI units [13]. In addition, there is a need to promote training in UGI endoscopy and functional studies of the esophagus [2].

Standardizing surgical training in UGI surgery is crucial. To address the current gaps, promoting a structured and incremental teaching approach can help residents to feel more confident and better understand the responsibilities involved in daily surgical practice. With adequate training and guidance, residents can eventually gain the ability to safely perform complex procedures [14–16]. Still, there is a lack of objective and robust data describing the operative experience of general surgical trainees outside the USA [17]. A previous national French survey study showed that a master diploma did not contribute to improve surgical training, whereas progressing in surgical autonomy was a driving factor to dissipate the feeling of dissatisfaction perceived by residents [18]. A worldwide survey focused on oncological UGI surgery showed that trainees spend much of their time observing or assisting particularly in esophageal resection, and more than half of the participants performed less than five UGI surgical procedures both in their residency and fellowship periods [10].

In the current era of “vocational crisis” in surgery and evolving technological scenario, it is important to provide more opportunities to improve the degree of satisfaction of the junior residents who should be encouraged to participate to regular pelvic trainer sessions, animal, and cadaver labs, and online video sessions. With the rising adoption of robotic surgery across Europe, standardized curricula will be fundamental for optimal training and certification, ensuring safety and consistent outcomes with this technology [19]. Also, in the near future, robotics and artificial intelligence with computer vision systems will likely enable residents to improve their technical skills and the quality of the surgical procedure by providing identification of anatomical landmarks and recognition of sequential steps of each surgical procedure [20, 21]. International guidelines and expert recommendations should shape the training pathway in this emerging scenario, emphasizing hands-on experience as a core component. However, there is a shortage of dedicated centers for such complementary training. Collaborative efforts among European experts, guided by the European Foregut Society (EFS) and the European association for Endoscopic Surgery (EAES), should create a comprehensive map of training centers that offer structured, hands-on educational opportunities. Lastly, the European Board of Surgery (FEBS) should certificate a minimum level of proficiency ensuring that recipients of the fellowship possess both the theoretical knowledge and practical skills necessary to excel in UGI surgery across Europe and beyond.

We recognize that the primary limitation of this study is the small sample size and the possible selection and recall bias. However, the present research serves as a cross-sectional overview of the European surgical education system and is intended to lay the foundation for developing dedicated guidelines for general surgical residency programs. Identifying gaps is beneficial only when efforts are aimed at finding solutions. Developing a competency-based curriculum alongside a multidisciplinary approach is vital for surgical residents. Expertise in surgery is achieved through practice, making high-quality education and training essential for adequately preparing the next generation of UGI surgeons and mentors [22–24].

## Conclusion

This survey highlights existing gaps in the educational programs for UGI surgery residents throughout Europe. Developing specific guidelines and recommendations could greatly enhance education and training. Residents should be involved at an early stage in all aspects of patients’ assessment and management. Extracurricular training courses should be available to fulfill the training needs and to provide appropriate opportunities for residents interested in UGI surgery. In the near future, transition from laparoscopy to robotics and evolution in deep learning and artificial intelligence are expected to change the scenario of surgical training by developing new models of apprenticeships.

## Supplementary Information

Below is the link to the electronic supplementary material.

Supplementary file1 (PDF 189 KB)

Supplementary file2 (PDF 183 KB)

## Data Availability

All raw data are available if required.
