# Supplementary material for: Education and training of surgical residents in upper gastrointestinal surgery: a European survey
Source: Updates Surg. 2025 Aug 16;78(1):55–62. doi: 10.1007/s13304-025-02362-3 (PMC12909444; doi:10.1007/s13304-025-02362-3)
Supplement: Supplementary file 1 — Supplementary file1 (PDF 189 KB) [file 13304_2025_2362_MOESM1_ESM.pdf]

International Survey: The involvement of surgical residents in the management of the foregut disease – The trainee's point of view

The European Foregut Society developed and promotes this survey in order to highlight the current problems of the training program of surgical residents in the field of upper GI surgery. This survey is dedicated to trainees and their opinion and experience regarding their training opportunities.

Section 1 of 7

This section gathers demographic and training context information.

E-mail

Age

Gender

Type of institution

- Academic hospital - General hospital

Country

Year of residency

Do you have the possibility to do your training in a dedicated upper GI surgery center?

- Yes / No

Section 2 of 7 - Available study materials & methods

This section of the survey is dedicated to assess the availability of theoretical materials, courses and events for studying upper gastrointestinal pathologies. Please respond with referring towards upper GI surgery related activities only.

Do you feel like there are enough materials available?

- Not at all — 1 — 2 — 3 — 4 — 5 — Extensive options

Do you have available structured theoretical courses?

- Not at all — 1 — 2 — 3 — 4 — 5 — Extensive options available

Do you have available video materials?

- Not at all — 1 — 2 — 3 — 4 — 5 — Extensive options

Rate the quality of the available study materials

- Poor — 1 — 2 — 3 — 4 — 5 — Excellent

What type of courses would you prefer?

- Dedicated to benign disease
- Dedicated to malignant disease
- Integrated benign + malignant disease
- Dedicated to a specific pathology

Do you feel the need of a structured course dedicated to upper GI surgery?

- No, not at all — 1 — 2 — 3 — 4 — 5 — Yes, definitely

Section 3 of 7 - The preoperative evaluation of the patient

This section analyzes involvement of surgical residents in preoperative patient evaluation.

How is the cooperation between GI department and surgery department?

- Totally independent — 1 — 2 — 3 — 4 — 5 — Excellent cooperation

Are surgeons in your department performing the preoperative investigations?

- Never — 1 — 2 — 3 — 4 — 5 — All the time

Do you, as residents, have the opportunity to assist to the preoperative management of the patient?

- Yes / No / Sometimes

Are you directly involved in any of the following investigations? (Yes / No / Sometimes)

- Endoscopy

- Manometry

- Ph/impedance testing

Have you performed any of the following investigations? (Yes / No / Sometimes)

- Endoscopy

- Manometry

- Ph/Impedance testing

Do you have any possibility to get dedicated training in any of the following investigations? (Yes / No / Maybe)

- Endoscopy

- Manometry

- Ph/impedance testing

Would you wish to be more involved in the preoperative part of the patient's management?

- No, I do not think this is needed — 1 — 2 — 3 — 4 — 5 — Yes, definitely!

Which skills do you believe should need more training?

- Endoscopy
- Manometry

- Ph/impedance testing
- Imaging studies

What kind of training opportunities would you wish for? (Open answer)

Section 4 of 7 - Intraoperative skills

The following section intends to evaluate the involvement of surgical residents in different upper gastrointestinal interventions.

Have you performed any upper GI surgical intervention as a first hand surgeon?

- Yes / No

How many?

Which interventions?

- Hiatal hernia repair
- Anti-reflux procedure
- Esophageal diverticulum
- Achalasia
- Esophagectomy
- Gastrectomy – subtotal
- Gastrectomy - total

Hiatal hernia repair - how many interventions have you assisted?

Hiatal hernia repair - Which of the following surgical steps have you performed yourself?

- Crura dissection
- Gastric fundus mobilization
- Cruroraphy
- Mesh placement
- Partial fundoplication
- Total fundoplication

Hiatal hernia repair - Which surgical steps do you find the most difficult to master?

- Crura dissection
- Gastric fundus mobilization
- Cruroraphy
- Mesh placement
- Partial fundoplication
- Total fundoplication

Hiatal hernia repair - Would you be confident in performing any of these surgical steps on your own?

- Crura dissection
- Gastric fundus mobilization
- Cruroraphy
- Mesh placement

- Partial fundoplication
- Total fundoplication

Esophagectomy - how many interventions have you assisted?

Esophagectomy - which of the following surgical steps have you performed yourself?

- Abdominal esophagus dissection
- Gastric mobilization
- Gastric conduit creation
- Thoracic esophagus dissection
- Cervical esophagus dissection
- Anastomosis

Esophagectomy - Which surgical steps do you find the most difficult to master?

- Abdominal esophagus dissection
- Gastric mobilization
- Gastric conduit creation
- Thoracic esophagus dissection
- Cervical esophagus dissection
- Anastomosis

Esophagectomy - Would you be confident in performing any of these surgical steps on your own?

- Abdominal esophagus dissection
- Gastric mobilization
- Gastric conduit creation
- Thoracic esophagus dissection
- Cervical esophagus dissection
- Anastomosis

Gastrectomy - how many interventions have you assisted?

Gastrectomy - which of the following surgical steps have you performed yourself?

- Lesser curvature dissection
- Greater curvature dissection
- Lymphadenectomy
- Reconstruction - subtotal gastrectomy
- Reconstruction – total gastrectomy

Gastrectomy - Which surgical steps do you find the most difficult to master?

- Lesser curvature dissection
- Greater curvature dissection

- Lymphadenectomy
- Reconstruction - subtotal gastrectomy
- Reconstruction – total gastrectomy

Gastrectomy - Would you be confident in performing any of these surgical steps on your own?

- Lesser curvature dissection
- Greater curvature dissection
- Lymphadenectomy
- Reconstruction - subtotal gastrectomy
- Reconstruction – total gastrectomy

Do you use intraoperative endoscopy for the following indications? (Yes / No / Sometimes)

- Hiatal hernia repair/ Anti-reflux surgery

- Heller miotomy

- Anastomosis verification

Are you familiar with endoscopic operative procedures? (Yes / No / Somewhat)

- Endo vacuum

- Esophageal stents

- Stricture dilatation

- POEM

- Flexible endoscopic treatment - Zenker diverticulum

- Rigid endoscopy - Zenker diverticulum

Do you have the possibility to train or practice these procedures? (Yes / No / Somewhat)

- Endo vacuum

- Esophageal stents

- Stricture dilatation

- POEM

- Flexible endoscopic treatment - Zenker diverticulum

- Rigid endoscopy - Zenker diverticulum

Section 5 of 7 - Postoperative follow-up

This section will refer to the postoperative management of the patient. Please respond with referring towards upper GI surgery related activities only.

Are you involved in the postoperative follow-up of patients? (Yes / No / Sometimes)

- Early postoperative follow-up

- Intensive care unit follow-up

- Late postoperative follow-up

Are you involved in the treatment of postoperative complications? (Yes / No / Sometimes)

- Endoscopic

- Surgical

Would you feel confident to manage the postoperative course of the patient by yourself?

- Not really — 1 — 2 — 3 — 4 — 5 — Yes, no problem!

What gaps do you feel you have in your practice? (Open answer)

Section 6 of 7 - Extracurricular activities

This section will focus on the availability and interest in any extracurricular activities. Please respond with referring towards upper GI surgery related activities only.

Do you have any extracurricular dedicated training opportunities in your center?

- No options — 1 — 2 — 3 — 4 — 5 — A lot of options

Are you interested in joining extracurricular activities?

- Not at all — 1 — 2 — 3 — 4 — 5 — Very interested

What kind of activities do you prefer?

- Conference/ Congress
- Webinar
- Hands-on training
- Live surgery
- Fellowship

What type of activities would you prefer to attend?

- National
- International

Regarding workshops - would you prefer to attend dedicated activities or workshops associated with conferences/congresses?

- Dedicated workshop
- Workshop associated to congress

What type of hands-on workshops do you believe are the most useful?

- Synthetic materials
- Ex-vivo biologic models
- Live animal surgery
- Simulators

Have you participated to any webinar/workshop/conference in the last year, dedicated to upper gastrointestinal surgery?

- Yes / No

If yes, did you feel it helped your practice?

- Not at all — 1 — 2 — 3 — 4 — 5 — Yes, very much

Are you encouraged to participate in this kind of activities?

- Not really — 1 — 2 — 3 — 4 — 5 — Very much

Are you involved in scientific activities? (writing articles, performing literature reviews, involvement in research activities)

- Not at all — 1 — 2 — 3 — 4 — 5 — All the time

Are you aware of the steps that need to be taken in order to conduct a research project?

- Not at all — 1 — 2 — 3 — 4 — 5 — I am familiar with it

In what type of scientific activities are you involved?

- Writing study protocols
- Data gathering
- Retrospective data studies
- Prospective data studies
- Clinical trials
- Literature reviews
- Writing scientific articles

Does your center emphasize the importance of scientific work in foregut surgery (reviewing up to date medical literature, performing research, being involved in research)?

- Not at all — 1 — 2 — 3 — 4 — 5 — Yes, it is a very important topic

How involved do you think the EFS (European Foregut Society) should be in training activities?

- Not at all — 1 — 2 — 3 — 4 — 5 — Extensive involvement

Section 7 of 7 - Diverse

This section covers diverse aspects of the training programs.

Do you wish to follow a career in upper GI surgery?

- Yes / No / Maybe

How important do you find the relationship mentor - resident in the training progress?

- Not very important — 1 — 2 — 3 — 4 — 5 — The most important factor

How important are the following factors in choosing the upper GI surgery specialization? (Not at all / Somewhat important / Neutral / Very important / The most important)

- Good mentor

- Possibility of working in a dedicated upper GI center

- Interesting pathology

- Role models

In which year of the residency program do you believe the upper GI surgery rotation is the most useful?

- 1 – 2 – 3 – 4 – 5 – 6

What is your perception of a career in upper GI surgery? (Not at all / Somewhat / Neutral / Pretty much / Very much)

- Time consuming

- Interesting pathologies

- Demanding surgical techniques

- International societies/collaboration

- Local experienced centers

- Prospect of a job

Is there a dedicated curriculum regarding mandatory skills to perform in foregut surgery during residency?

- Yes / No / I do not know
