# Supplementary material for: Education and training of surgical residents in upper gastrointestinal surgery: a European survey
Source: Updates Surg. 2025 Aug 16;78(1):55–62. doi: 10.1007/s13304-025-02362-3 (PMC12909444; doi:10.1007/s13304-025-02362-3)
Supplement: Supplementary file 2 — Supplementary file2 (PDF 183 KB) [file 13304_2025_2362_MOESM2_ESM.pdf]

International Survey: The involvement of surgical residents in the management of the foregut disease - The trainer's point of view

The European Foregut Society developed and promotes this survey in order to highlight the current problems of the training program of surgical residents in the field of upper GI surgery. This survey is dedicated to the trainers and their opinion and experience regarding the training opportunities of the surgical residents.

Section 1 of 7

This section gathers demographic and training coordination information from trainers.

E-mail

Age

Gender

Type of institution

- Academic hospital - General hospital

Country

How many residents do you coordinate per year?

Do you work in a dedicated upper GI department?

- Yes / No

How many upper GI surgical interventions do you perform in your center per year? (< 20 / 20-50 / 50-100 / >100)

- Esophagectomy

- Gastrectomy

- Anti-reflux surgery + Hiatal hernia repair

Section 2 of 7 - Available study materials & methods

This section of the survey is dedicated to assess the availability of theoretical materials, courses and events for studying upper gastrointestinal pathologies. Please respond with referring towards upper GI surgery related activities only.

Do you feel like there are enough materials available?

- Not at all — 1 — 2 — 3 — 4 — 5 — Extensive options

Do you have available structured theoretical courses?

- Not at all — 1 — 2 — 3 — 4 — 5 — Extensive options

Do you have available video materials?

- Not at all — 1 — 2 — 3 — 4 — 5 — Extensive options

Rate the quality of the available study materials

- Poor — 1 — 2 — 3 — 4 — 5 — Excellent

Do you feel that your trainees might need a structured course dedicated to upper GI surgery?

- No, not at all — 1 — 2 — 3 — 4 — 5 — Yes, definitely

Would you be interested to contribute to a network of upper GI surgery training?

- Yes / No / Maybe

What type of courses would you prefer?

- Dedicated to benign disease
- Dedicated to malignant disease
- Integrated benign + malignant disease
- Dedicated to a specific pathology

If yes, what type of contributions would you prefer?

- Operative videos
- Prerecorded lectures
- Live lectures
- Others

Section 3 of 7 - The preoperative evaluation of the patient

This section intends to analyze the involvement of surgical residents in the preoperative management of the patient.

How is the cooperation between GI department and surgery department?

- Totally independent — 1 — 2 — 3 — 4 — 5 — Excellent cooperation

Are surgeons in your department performing the preoperative investigations?

- Never — 1 — 2 — 3 — 4 — 5 — All the time

Do your residents have the opportunity to assist to the preoperative management of the patient?

  - Yes / No / Sometimes

Are the residents directly involved in any of the following investigations? (Yes / No / Sometimes)

- Endoscopy

- Manometry

- Ph/impedance testing

Do your residents perform any of the following investigations? (Yes / No / Sometimes)

- Endoscopy

- Manometry

- Ph/Impedance testing

Do your residents have any possibility to get dedicated training in any of the following investigations? (Yes / No / Maybe)

- Endoscopy

- Manometry

- Ph/impedance testing

Would you wish that your residents would be more involved in the preoperative part of the patient's management?

- No, I do not think this is useful — 1 — 2 — 3 — 4 — 5 — Yes, definitely!

Which skills do you believe should need more training?

  - Endoscopy

- Manometry
- Ph/impedance testing
- Imaging studies

What kind of training opportunities would you wish for your residents? (Open answer)

Section 4 of 7 - Intraoperative skills

The following section intends to evaluate the involvement of surgical residents in different upper gastrointestinal interventions.

Did your residents perform an entire upper GI surgical intervention as a first hand surgeon, under supervision?

- Yes / No

How many? (per year)

Which interventions?

- Hiatal hernia repair
- Anti-reflux procedure
- Esophageal diverticulum
- Achalasia
- Esophagectomy
- Gastrectomy – subtotal
- Gastrectomy - total

If not, please list the reasons why:

- I do not have the trust that they can manage such an intervention
- I find it too risky
- I believe they need more training
- I find it unethical
- I do not think it's useful for their training

Hiatal hernia repair - Which of the following surgical steps do the residents perform by their own?

- Crura dissection
- Gastric fundus mobilization
- Cruroraphy
- Mesh placement
- Partial fundoplication
- Total fundoplication

Hiatal hernia repair - Which surgical steps do you find the most difficult to master?

- Crura dissection
- Gastric fundus mobilization
- Cruroraphy
- Mesh placement
- Partial fundoplication

- Total fundoplication

Hiatal hernia repair - Would you be confident to let your residents perform any of these surgical steps on their own?

- Crura dissection
- Gastric fundus mobilization
- Cruroraphy
- Mesh placement
- Partial fundoplication
- Total fundoplication

Esophagectomy - which of the following surgical steps do the residents perform by their own?

- Abdominal esophagus dissection
- Gastric mobilization
- Gastric conduit creation
- Thoracic esophagus dissection
- Cervical esophagus dissection
- Anastomosis

Esophagectomy - Which surgical steps do you find the most difficult to master?

- Abdominal esophagus dissection
- Gastric mobilization
- Gastric conduit creation
- Thoracic esophagus dissection
- Cervical esophagus dissection
- Anastomosis

Esophagectomy - Would you be confident to let your residents perform any of these surgical steps on their own?

- Abdominal esophagus dissection
- Gastric mobilization
- Gastric conduit creation
- Thoracic esophagus dissection
- Cervical esophagus dissection
- Anastomosis

Gastrectomy - Which of the following surgical steps do the residents perform by their own?

- Lesser curvature dissection
- Greater curvature dissection

- Lymphadenectomy
- Reconstruction - subtotal gastrectomy
- Reconstruction – total gastrectomy

Gastrectomy - Which surgical steps do you find the most difficult to master?

- Lesser curvature dissection
- Greater curvature dissection
- Lymphadenectomy
- Reconstruction - subtotal gastrectomy
- Reconstruction – total gastrectomy

Gastrectomy - Would you be confident to let your residents perform any of these surgical steps on their own?

- Lesser curvature dissection
- Greater curvature dissection
- Lymphadenectomy
- Reconstruction - subtotal gastrectomy
- Reconstruction – total gastrectomy

Do you use intraoperative endoscopy for the following indications? (Yes / No / Sometimes)

- Hiatal hernia repair/ Anti-reflux surgery

- Heller miotomy

- Anastomosis verification

Do your residents have the possibility to train or practice the following operative endoscopy procedures? (Yes / No / Somewhat)

- Endo vacuum

- Esophageal stents

- Stricture dilatation

- POEM

- Flexible endoscopic treatment - Zenker diverticulum

- Rigid endoscopy - Zenker diverticulum

Section 5 of 7 - Postoperative follow-up

This section will refer to the postoperative management of the patient. Please respond with referring towards upper GI surgery related activities only.

Are your residents involved in the postoperative follow-up of patients? (Yes / No / Sometimes)

- Early postoperative follow-up

- Intensive care unit follow-up

- Late postoperative follow-up

Are your residents involved in the treatment of postoperative complications? (Yes / No / Sometimes)

- Endoscopic

- Surgical

Would you feel confident to allow your residents to manage the postoperative course of the patient by themselves?

- Not really — 1 — 2 — 3 — 4 — 5 — Yes, no problem!

What gaps do you feel your residents have in their practice? (Open answer)

Section 6 of 7 - Extracurricular activities

This section will focus on the availability and interest in any extracurricular activities. Please respond with referring towards upper GI surgery related activities only.

Do you have any extracurricular dedicated training opportunities in your center?

- No options — 1 — 2 — 3 — 4 — 5 — A lot of options

Are your residents interested in joining extracurricular activities?

- Not at all — 1 — 2 — 3 — 4 — 5 — Very interested

What kind of activities do you prefer?

- Conference/ Congress
- Webinar
- Hands-on training
- Live surgery
- Fellowship

Did your residents participate to any webinar/workshop/conference in the last year, dedicated to upper gastrointestinal surgery?

- Yes / No

If yes, did you feel it helped their practice?

- Not at all — 1 — 2 — 3 — 4 — 5 — Yes, very much

Do you encourage them to participate in this kind of activities?

- Not really — 1 — 2 — 3 — 4 — 5 — Very much

Are your residents involved in scientific activities? (writing articles, performing literature reviews, involvement in research activities)

- Not at all — 1 — 2 — 3 — 4 — 5 — All the time

How involved do you think the EFS (European Foregut Society) should be in training activities?

- Not at all — 1 — 2 — 3 — 4 — 5 — Extensive involvement

Section 7 of 7 - Diverse

This last section covers diverse aspects of the training program.

How important do you find the relationship mentor - resident in the training progress?

- Not very important — 1 — 2 — 3 — 4 — 5 — The most important factor

In which year of the residency program do you believe the upper GI surgery rotation is the most useful?

- 1 – 2 – 3 – 4 – 5 – 6

Is there a dedicated curriculum regarding mandatory skills to perform in foregut surgery during residency?

- Yes / No / I do not know
